# Evidence of Differences in Cellular Regulation of *Wolbachia*-Mediated Viral Inhibition between Alphaviruses and Flaviviruses

**DOI:** 10.3390/v16010115

**Published:** 2024-01-13

**Authors:** Stephanie M. Rainey, Daniella A. Lefteri, Christie Darby, Alain Kohl, Andres Merits, Steven P. Sinkins

**Affiliations:** 1 MRC-University of Glasgow-Centre for Virus Research, Garscube Campus, University of Glasogw, Glasgow G61 1QH, UK; daniella.lefteri@glasgow.ac.uk (D.A.L.); alain.kohl@lstmed.ac.uk (A.K.); 2 Departments of Tropical Disease Biology and Vector Biology, Liverpool School of Tropical Medicine, Liverpool L3 5QA, UK; 3 Institute of Technology, University of Tartu, 50411 Tartu, Estonia; andres.merits@ut.ee

**Keywords:** *Wolbachia*, alphaviruses, flaviviruses

## Abstract

The intracellular bacterium *Wolbachia* is increasingly being utilised in control programs to limit the spread of arboviruses by *Aedes* mosquitoes. Achieving a better understanding of how *Wolbachia* strains can reduce viral replication/spread could be important for the long-term success of such programs. Previous studies have indicated that for some strains of *Wolbachia*, perturbations in lipid metabolism and cholesterol storage are vital in *Wolbachia*-mediated antiviral activity against the flaviviruses dengue and Zika; however, it has not yet been examined whether arboviruses in the alphavirus group are affected in the same way. Here, using the reporters for the alphavirus Semliki Forest virus (SFV) in *Aedes albopictus* cells, we found that *Wolbachia* strains *w*Mel, *w*Au and *w*AlbB blocked viral replication/translation early in infection and that storage of cholesterol in lipid droplets is not key to this inhibition. Another alphavirus, o’nyong nyong virus (ONNV), was tested in both *Aedes albopictus* cells and in vivo in stable, transinfected *Aedes aegypti* mosquito lines. The strains *w*Mel, *w*Au and *w*AlbB show strong antiviral activity against ONNV both in vitro and in vivo. Again, 2-hydroxypropyl-β-cyclodextrin (2HPCD) was not able to rescue ONNV replication in cell lines, suggesting that the release of stored cholesterol caused by *w*Mel is not able to rescue blockage of ONNV. Taken together, this study shows that alphaviruses appear to be inhibited early in replication/translation and that there may be differences in how alphaviruses are inhibited by *Wolbachia* in comparison to flaviviruses.

## 1. Introduction

Arboviruses, or viruses spread by arthropods, account for an estimated 17% of global communicable infections [1]. As there are few treatments and vaccines available for such diseases, vector control measures are usually implemented to lower vector numbers or reduce vector competence. One such measure is the introduction of the endosymbiotic bacterium *Wolbachia pipientis*, which has been shown to dramatically reduce the ability of *Aedes* species to spread arboviruses such as dengue when non-native strains are introduced. Release programs have shown that *Ae. aegypti* carrying the *w*Mel or *w*AlbB strain of *Wolbachia* are able to rapidly and stably replace native populations and considerably reduce the incidence of dengue [2], in Malaysia for *w*AlbB and Indonesia and Brazil for *w*Mel [3,4]. The latter study also showed a decrease in the incidence of the alphavirus chikungunya virus (CHIKV). *Wolbachia* therefore is a cost-effective and efficient control measure against arboviruses spread by *Aedes* mosquitoes.

Although the effectiveness of *Wolbachia* release programs has been shown, the mechanism by which *Wolbachia* is able to inhibit virus transmission and therefore reduce vector competence is not yet fully understood. To date, *Wolbachia* has been shown to be effective at reducing levels of virus in cells and mosquitoes from several positive-sense RNA virus genera including both flaviviruses such as dengue (DENV) and Zika (ZIKV) and alphaviruses such as CHIKV and Semliki Forest virus (SFV). However, it has not been established if *Wolbachia* blocks all viruses through the same mechanism(s).

Studies have indicated that blockage of viral replication/translation occurs early after infection, that entry is not involved and that the origin of viral RNA is not important, suggesting that competition for cellular resources and/or compartments, required early in infection, is a crucial factor [5]. RNA binding proteins have been indicated in the *Wolbachia*-mediated antiviral activity against both flaviviruses and alphaviruses [6,7,8,9]. Changes in amino acid availability, transcriptome and autophagy caused by *Wolbachia* have also been implicated as likely mechanisms [10,11].

Both alphaviruses and flaviviruses require reassortment and availability of lipids, lipid membranes and cholesterol for several aspects of their lifecycle in both mammalian and mosquito cells. Perturbations in such pathways have been shown to be involved in *Wolbachia*-mediated antiviral activity against flaviviruses. The ability of the strains *w*Mel and *w*MelPop to block flaviviruses DENV and ZIKV in both *Aedes aegypti* and *Aedes albopictus* is at least partially due to changes in cholesterol dynamics and lipid metabolism [12,13,14]. It has been shown that strain *w*Au does not cause similar perturbations in *Aedes* cells/mosquitoes, and it is likely that they do not play an important role in the ability of *w*Au to block arbovirus replication [13].

It is therefore evident that more research is required to fully understand the mechanism(s) behind *Wolbachia*-mediated antiviral activity and to determine if all strains of *Wolbachia* block arbovirus infection using similar mechanism(s), if all viruses are blocked by the same mechanism(s) and if such mechanism(s) are the same across different mosquito species. This study set out to determine if alphavirus replication/translation is blocked early in infection and if cholesterol perturbation plays a role in alphavirus inhibition in an *Aedes albopictus* model cell system including three different strains of *Wolbachia*. Alphaviruses offer a unique opportunity to study the early stages of viral replication/translation, as their genomes contain two viral promoters: a genomic and subgenomic. Once infection takes place, an initial round of translation occurs from the incoming positive-sense genome, producing the non-structural proteins representing subunits of the replicase. This then allows for a further round of replication to take place, producing a full-length anti-genome, giving rise to the availability of the subgenomic promotor from which mRNA for structural proteins is expressed [15,16,17,18,19]. Therefore, reporter viruses, replicons and trans-replicase systems containing reporter constructs can be utilised to unpick different stages of the viral lifecycle [5,16,20,21,22]. In this study, such systems were utilised to study the effect of *Wolbachia* on two distinct alphaviruses ONNV and SFV.

## 2. Materials and Methods

### 2.1. Mosquito Rearing and Cell Work

Cells have been previously described [13,23]. Briefly, Aa23 *Ae. albopictus* cells, naturally infected with *w*AlbB, cleared of *Wolbachia* or transinfected with *w*Mel or *w*Au, were maintained at 28 °C in 25 cm^2^ flasks in Schneider’s *Drosophila* media (Pan Bioscience, Cambridge, UK) containing 10% heat-inactivated FBS (Thermo Fisher, London, UK).

Baby Hamster Kidney (BHK-21s) cells were maintained at 37 °C with 5% CO_2_ in Glasgow MEM media supplemented with 5% FBS, 1% Penicillin/streptomycin and 10% Tryptose Phosphate Buffer (Thermo Fisher, UK). Cells were split twice weekly at a ratio of 1:10.

Mosquito colonies have been described previously [12,24,25]. Colonies were maintained at standard 27 °C and 70% relative humidity with a 12 h light/dark cycle. All lines were created from a standard *Wolbachia*-free background. Larvae were fed with tropical fish food pellets (Tetramin, Tetra, Melle, Germany), and adults were provided with sugar meals ad libitum. For bloodmeals, females were provided human blood (Scottish Blood Bank) via a Hemotek artificial blood feeding system (Hemotek, Blackburn, UK). After mating, damp filter paper (Grade 1 filter paper, Whatman plc, GE healthcare, Buckinghamshire, UK) was provided as egg cones, and females were allowed to oviposition. Egg cones were collected and desiccated for 5–10 days prior to hatching in water containing 1 g/L of Bovine liver powder (MP Biomedicals, Santa Ana, CA, USA).

### 2.2. Viruses and Viral Clones

SFV4 expressing *FFLuc* has been described previously [26]. The plasmid containing corresponding infectious cDNA (icDNA), pCMV-SFV4(3H)-*FFLuc*, was electroporated into BHK-21 cells, growth media containing 2% FBS was added, and cells were incubated at 37 °C until clear CPE was detected. Supernatant was clarified by centrifugation and aliquoted for storage at −80 °C. Virus was titred via plaque-forming assays as described previously [27]. ONNV-2SG-ZsGreen was created from the icDNA of ONNV Chad strain with ZsGreen cloned between native and duplicated SG promoters of ONNV. Plasmids containing icDNA of ONNV-2SG-ZsGreen were linearised with PmeI (New England BioLabs), transcribed in vitro utilising a MEGAscript SP6 polymerase kit (Ambion); obtained capped RNAs were transfected into BHK-21 cells as described previously [20].

SFV1(3F)R*Luc*-SG-*FFluc* replicon has been described previously [5]. Briefly, pSFV1(3F)*RLuc*-SG-*FFLuc* plasmid was linearised with SpeI and purified with Qiagen PCR purification kit (Qiagen, UK). A total of 1 μg of DNA was then in vitro transcribed to produce viral-replicon RNA utilising a MEGAscript SP6 polymerase kit (Ambion) and used as described below.

The SFV trans-replicase system has been described previously [5]. For this study, the *Drosophila* Actin promoter was replaced with the *Ae. aegypti* PUb promoter sequence for optimal expression in mosquitoes [28].

### 2.3. Infection and Transfection of Viruses and Viral Replicon/Trans-Replicases

For viral infections, 5.5 × 10^5^ cells were plated in 24-well plates and left to adhere overnight. Cells were then treated or used directly for infection. For (2HPCD) experiments, cells were plated, and after incubation for 24 h, PBS or 2HPCD (Merck, London, UK) was added to the media at differing concentrations, and cells were further incubated for 48 h. Media was then removed, and cells were gently washed in PBS before ONNV or SFV was added at 0.1 MOI. For direct infection, each virus (at an MOI 0.1) was added directly to cells after 24 h; after 1 h, media was removed and replaced with fresh-FBS-supplemented media. At 24, 48 and/or 72 hpi (as stated in figures), supernatant was removed and either kept for assessment via plaque assay as described above or discarded. A total of 500 μL of Trizol (Thermo Fisher, UK) was added to cells, and RNA and DNA were extracted per manufacturer’s protocol. Alternatively, cells were lysed with passive lysis buffer (Promega, London, UK).

Transfection of viral-replicon RNA or trans-replicase plasmids was carried out as previously described with the following change: lipofectamine 3000 (Thermo Fisher, UK) was used as transfection reagent according to manufacturer’s protocol.

For in vivo infections of ONNV, 4–5-day-old female mosquitoes were provided with an infectious bloodmeal containing 1 × 10^7^ PFU/mL of virus. Twelve days post infection, mosquitoes were anesthetised on ice, and salivary glands were dissected. Glands were placed in GMEM and used for plaque assay assessment. The remaining carcass was placed in 500 μL of Trizol, and RNA/DNA was extracted as per manufacturer’s protocol.

### 2.4. qPCR and Luciferase Assay for Measurement of Virus

Aa23 cells (Aa23*w*AlbB, Aa23*w*Au, Aa23*w*Mel and Aa23) infected with virus (SFV4(3H)-*FFLuc*), transfected with viral RNA (SFV1(3F)R*Luc*-SG-*FFLuc*) or transfected with SFV trans-replicase plasmids were lysed as stated above in passive lysis buffer. Detection of luciferase expression was performed using dual luciferase assay activity kit (Promega, UK) as previously described [5].

RNA and DNA from Aa23 cells (Aa23*w*AlbB, Aa23*w*Au, Aa23*w*Mel and Aa23) and *Ae. aegypti* mosquitoes (wildtype, *w*Mel, *w*Au and *w*AlbB) infected with ONNV virus were utilised to measure viral RNA, and *Wolbachia* DNA was carried out via RT-qPCR or qPCR as previously described [20,25].

### 2.5. Statistical Analysis

All statistical analyses were performed using GraphPad Prism version 10.0.0 for MacOS, GraphPad Software, Boston, MA, USA, www.graphpad.com (accessed on 10 December 2023). For data with normal distribution as calculated in Prism, an ordinary one-way ANOVA was carried out. For data that did not follow a normal distribution, the nonparametric Kruskal–Wallis test was carried out. All cell experiments consisted of 3 independent replicates carried out in duplicate except for 2HPCD, which consisted of 2 independent replicates carried out in duplicate.

## 3. Results

### 3.1. Wolbachia Blocks Early SFV Replication/Translation Events in Aedes albopictus Cells

**Figure 1:**
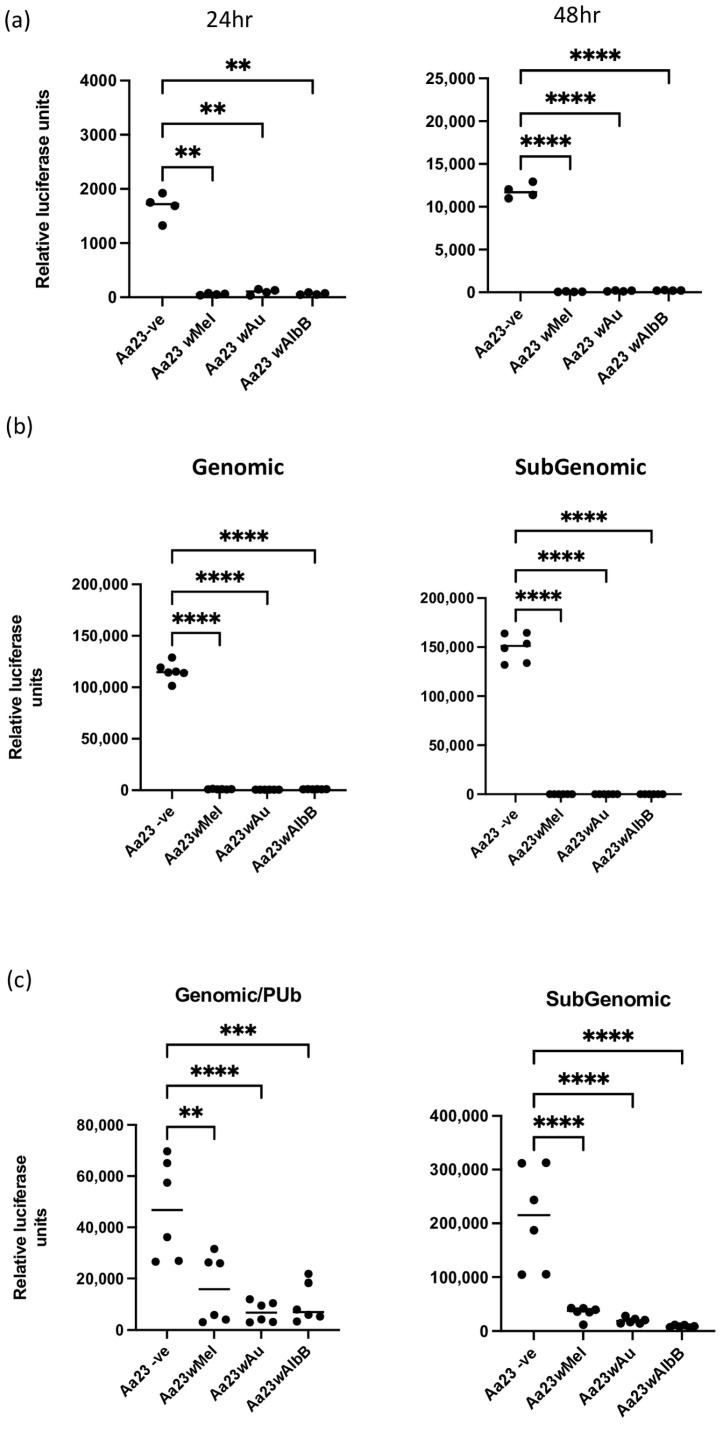
*Wolbachia* blocks early replication/translation of SFV in Aa23 cells. Aa23 cells cleared of *Wolbachia* or carrying *w*Mel, *w*AlbB or *w*Au strains were infected or transfected as follows: (**a**) Cells were infected with SFV4(3H)-*FFLuc* at an MOI of 0.1 and lysed at 24 and 48 hpi, and levels of SFV were measured via luciferase assay. Each graph shows 2 independent experiments carried out in duplicate. Statistical significance was determined via an ordinary one-way ANOVA. (**b**) Cells were transfected with in vitro transcribed SFV1(3F)R*Luc*-SG-*FFluc* RNA. At 24 h post transfection, cells were lysed, and both *Renilla* and Firefly luciferase activities were measured. Each graph shows three independent replicates carried out in duplicate. Y axis represents relative light units/55,000 cells. Statistical significance was determined via an ordinary one-way ANOVA. (**c**) Cells were transfected with two plasmids, one containing the viral template where *FFLuc* activity is under the control of the *Aedes* PUb promoter and SFV genomic promoter and *Gluc* is under the control of the SFV subgenomic promoter (template) and one containing the viral replicase region under PUb control (replicase). Y axis represents relative light units/55,000 cells. Each graph shows 3 independent experiments carried out in duplicate. Statistical significance was determined via an ordinary one-way ANOVA. Plots have statistical significance indicated as follows: ** *p* < 0.01, *** *p* < 0.001 and **** *p* < 0.0001.

*Ae. albopictus* (Aa23) cells naturally infected with *w*AlbB, transinfected with *w*Mel or *w*Au or cleared of *Wolbachia* were first infected with reporter virus SFV4(3H)-*FFLuc* [26], whereby luciferase expression acts a proxy for viral replication, at an MOI of 0.1. Luciferase activity and therefore viral replication were measured at 24 and 48 h post infection (hpi). In *Wolbachia*-cleared cells, luciferase activity was high at 24 hpi and increased by a log10 at 48 hpi, clearly showing viral replication. However, in cells containing any of the three *Wolbachia* strains, luciferase activity was significantly reduced to close to background levels at both time points, indicating strong inhibition of SFV replication/translation (Figure 1a).

To determine the likely stages at which *Wolbachia* blocks alphavirus replication/translation, we utilised a reporter replicon system previously developed for SFV, SFV1(3F)R*Luc*-SG-*FFluc* [5], where the expression of reporters is mediated either by the genomic promoter or subgenomic promoter of SFV. Compared to *Wolbachia*-cleared cells, luciferase activity from both the genomic (RLuc) and subgenomic (FFLuc) promoter was significantly lower in each of the three cell lines containing different *Wolbachia* strains (Figure 1b). These results indicate that all three strains of *Wolbachia* cause an early inhibition of replication/translation of RNAs produced from both promotors and that entry is unlikely to be important (entry is bypassed by the transfection of viral RNA).

As previous data indicated that uncoupling viral replicase expression from viral RNA replication through the introduction of replicase-expressing plasmids, together with a separate replication-competent template RNA (mini-genome), to cells via plasmid transfection did not overcome *w*Mel-mediated antiviral activity in *Drosophila* cells, the previously described SFV trans-replicase system [5] was utilised to determine if this was also the case in Aa23 cells and with other strains of *Wolbachia*. The *Drosophila* actin promoter was replaced with the constitutively active *Ae. aegypti* polyubiquitin promoter (PUb). Two plasmids, one containing the sequence encoding for SFV replicase under PUb and one containing FFLuc under the control of both PUb and the SFV genomic promoter and GLuc under the control of the SFV subgenomic reporter, were transfected into the Aa23 cell lines. Luciferase activity from both the genomic and subgenomic promoters was significantly reduced by all three strains of *Wolbachia*, indicating that the origin of viral RNA is not important in *Wolbachia* -mediated antiviral activity (Figure 1c).

### 3.2. Wolbachia-Mediated Antiviral Activity against SFV Is Not Rescued by the Addition of 2HPCD

**Figure 2:**
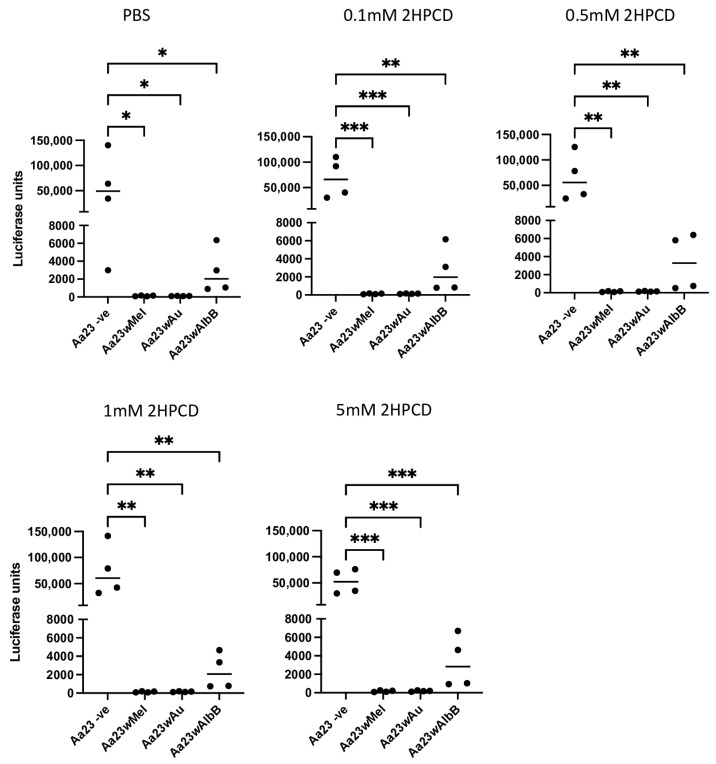
2HPCD does not rescue *Wolbachia*-mediated inhibition of SFV. Aa23 cells cleared of *Wolbachia* or carrying *w*Mel, *w*AlbB or *w*Au strains were treated with PBS or differing concentrations of 2HPCD for 48 h prior to infection with 0.1 MOI of SFV4(3H)-*FFLuc*. At 48 hpi, luciferase assays were carried out to determine levels of SFV4(3H)-*FFLuc.* Each graph shows 2 independent experiments carried out in duplicate. Y axis represents relative light units/55,000 cells. Statistical significance was determined via an ordinary one-way ANOVA. Plots have statistical significance indicated as follows: * *p* < 0.05, ** *p* < 0.01 and *** *p* < 0.001.

Previous data have shown that the ability of *w*Mel to block DENV and ZIKV replication in Aa23 cell lines is partially due to the perturbation of lipid and cholesterol metabolism. To determine if the treatment of *w*Mel cells with 2HPCD could rescue SFV infection, the three *Wolbachia* cell lines and the cleared control were treated for 48hr with differing concentrations of 2HPCD, before being infected with SFV4(3H)-*FFLuc*. At 48 hpi, cells were lysed, and luciferase activity was measured. In all three cell lines containing *Wolbachia,* 2HPCD was not able to rescue viral inhibition (Figure 2).

### 3.3. Wolbachia Strains Block ONNV Replication/Translation and Production of Infectious Particles in Aa23 Cells, and 2HPCD Does Not Rescue Viral Inhibition

**Figure 3:**
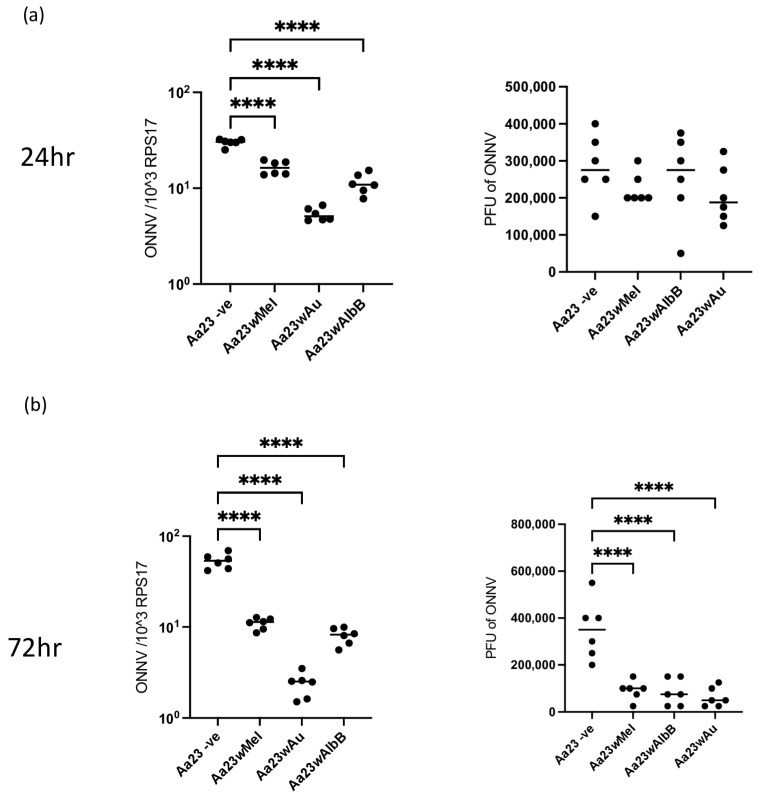
Time course of ONNV infection in Aa23 cells shows *Wolbachia* blocks replication and production of infectious particles. Aa23 cells cleared of *Wolbachia* or carrying *w*Mel, *w*AlbB or *w*Au strains were infected with ONNV virus (MOI 0.1). At (**a**) 24 and (**b**) 72 hpi, supernatant and cells were collected, and viral RNA levels in cells and viral titre in the supernatant were measured. Each graph shows 3 independent experiments carried out in duplicate. Statistical significance was determined via an ordinary one-way ANOVA. Plots have statistical significance indicated as follows: **** *p* < 0.0001.

**Figure 4:**
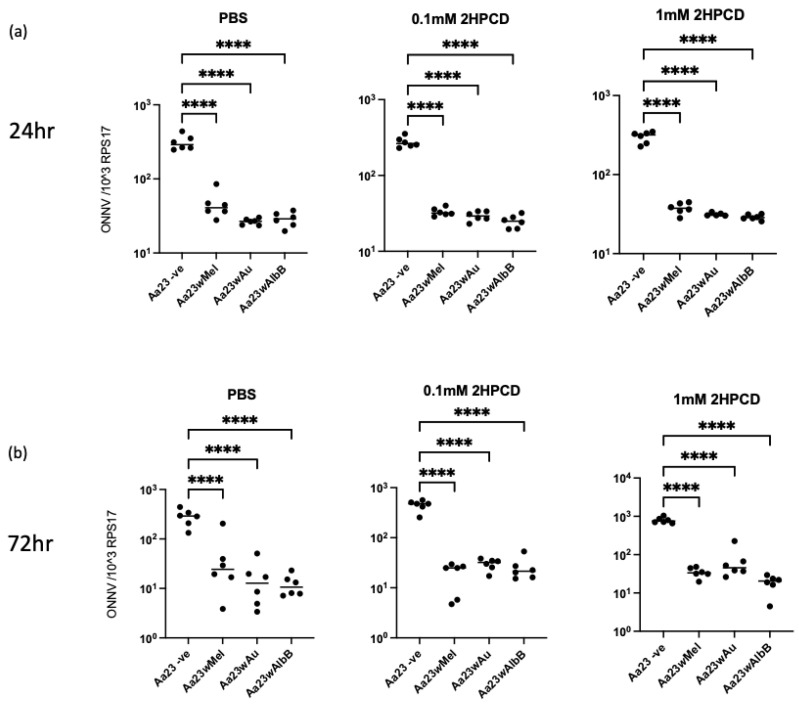
2HPCD does not rescue *Wolbachia* -mediated inhibition of ONNV virus. Aa23 cells cleared of *Wolbachia* or carrying *w*Mel, *w*AlbB or *w*Au strains were treated with PBS or differing concentrations of 2HPCD for 48 h prior to infection with ONNV at an MOI of 0.1. At 24 (**a**) and 72 (**b**) hpi, cells were collected, and level of viral RNA was measured by RT-qPCR. Each graph shows 3 independent experiments carried out in duplicate. Statistical significance was determined via an ordinary one-way ANOVA. Plots have statistical significance indicated as follows: **** *p* < 0.0001.

To determine if the antiviral activity of the three strains of *Wolbachia* extends to other alphaviruses, Aa23 cells were infected with ONNV-2SG-ZsGreen; after 24 and 72 hpi, viral RNA was measured using RT-qPCR, and infectious particles were measured via plaque assay. At 24 hpi, there was significantly less viral RNA present in cells containing *Wolbachia* when compared to cleared cells, with *w*Au showing the strongest inhibition. However, there was no significant difference observed in infectious particles. By 72 hpi, there were significantly lower levels of both viral RNA and infectious particles with all three strains of *Wolbachia*, again with *w*Au demonstrating the strongest inhibition (Figure 3). As for SFV, the addition of 2HPCD to cells was not able to rescue ONNV replication (Figure 4).

### 3.4. Stable Transinfection of wMel, wAlbB and wAu in Ae. aegypti Mosquitoes Limits ONNV Replication and Dissemination

**Figure 5:**
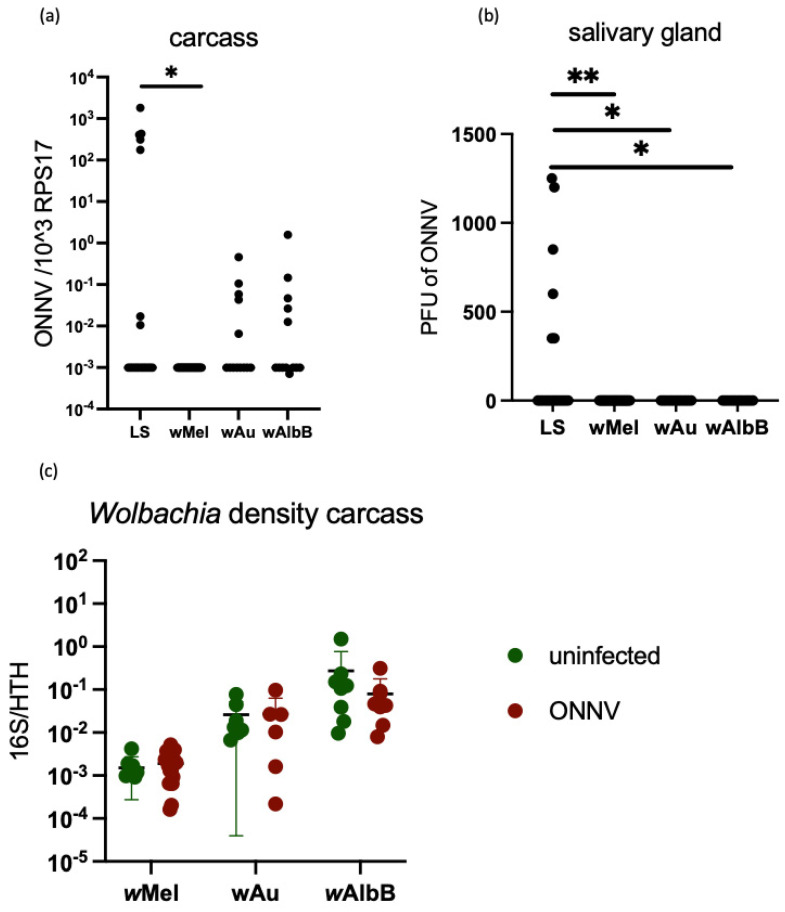
*Wolbachia* blocks dissemination of ONNV in vivo in *Aedes aegypti* mosquitoes. 4–5-day-old female mosquitoes were fed an infectious bloodmeal containing 1 × 10^7^ pfu/mL of ONNV or a non-infectious control bloodmeal. Twelve days post infection, salivary glands were dissected, and the remaining carcass was collected. The level of viral RNA in the carcass was measured via RT-qPCR, statistical significance was determined via Kruskal–Wallis test (**a**), and the level of dissemination of infectious virus to the salivary glands was measured via plaque assay; statistical significance was determined via Kruskal–Wallis test (**b**). The effect of ONNV on *Wolbachia* density: levels of *Wolbachia* were determined via qPCR in females fed with both an infectious and non-infectious bloodmeal (**c**). Plots have statistical significance indicated as follows: * *p* < 0.05 and ** *p* < 0.01.

To determine if the stable transinfection of *Wolbachia* in *Ae. aegypti* leads to the inhibition of ONNV infection, 4–5-day-old female mosquitoes stably transinfected with either *w*Mel, *w*Au or *w*AlbB and the native *Wolbachia*-free line (LS) were infected with ONNV via an infectious bloodmeal. Dissemination of infectious virus to the salivary glands and viral RNA levels in the remaining carcass were measured at 12 days post infection. 36.8% (7 of 19) of the lab strain (LS) *Wolbachia*-free LS line became infected with ONNV, and 31.5% (6 of 19) had infectious particles in the salivary glands (Figure 5a,b). Viral RNA was undetectable in *w*Mel mosquitoes, and both *w*AlbB and *w*Au showed a lower level of viral RNA than LS, although the difference was not significant (Figure 5a). Dissemination of infectious particles to the salivary glands was completely blocked by all three strains of *Wolbachia*, with no plaques detected via plaque assay (Figure 5b). ONNV infections had no effect on the density of *Wolbachia* within the mosquitoes (Figure 5c).

## 4. Discussion

Arboviruses continue to expand their global importance, and with climate change and vector invasive spread, outbreaks in new areas are inevitable. It is important therefore to determine the range of viruses blocked by different *Wolbachia* strains and also whether *Wolbachia* block viruses by the same mechanism(s). Previous data have shown that early viral replication/translation of SFV is inhibited by strain *w*Mel in *Drosophila* cells, indicating that competition for cellular resources and/or manipulation of cellular environments is key to *Wolbachia*-mediated antiviral activity, rather than the bacteria mounting a “response to viral replication” [5]. This study recapitulated these data and showed that in *Ae. albopictus* cell lines, three strains, *w*Mel, *w*Au and *w*AlbB, block SFV replication/translation; bypassing entry does not rescue replication, and supplying viral RNA via transfected plasmids does not lead to an increase in SFV replication. Interestingly, unlike results from Rainey et al. (2016), the expression of luciferase from the genomic promoter and the PUb promoter in the viral trans-replicase system was significantly reduced by all three strains of *Wolbachia*. This difference may be due to a difference in the strength of the promoters used; that is, PUb may result in a lower level of expression than the *Drosophila Actin* promoter, and therefore, the expression does not overwhelm the system [5,22]. Alternatively, the overall transfection efficiency may be affected by the presence of *Wolbachia*. However, in the absence of the replicase, luciferase activity solely under the control of PUb is not significantly different in *w*Au or *w*Mel cells when compared to cleared cells, suggesting that in these two cell lines, overall transfections are not affected (Supplementary Figure S1); however, overall effects on transfection cannot be ruled out in cells containing *w*AlbB. Alternatively, or in addition, it is possible that *Wolbachia* reduces the translation of the 5′ ends of the RNAs, which correspond to the beginning of the SFV genome. The same sequence, around 300 bp in length, is shared by the virus genome, the replicon vector and transcripts generated from the template plasmid. All three RNAs should express reporters in a replication-independent manner. For cells harboring *Wolbachia*, the expression of the reporter was, however, not observed if the cells were infected with SFV (Figure 1a) or transfected with replicon RNA (Figure 1b), and the reporter expression was reduced if the cells were transfected with template-RNA-expressing plasmids (Supplementary Figure S1), which may be explained by the inhibition of the 5′ ends of the RNA.

Previous data have indicated that lipid metabolism and cholesterol likely play a key role in the ability of *w*Mel to block both ZIKV and DENV infection in *Aedes* cells. Infection of both flaviviruses was restored in the presence of the cyclodextrin 2HPCD, which was shown to release trapped cholesterol from lipid droplets [12,13]. However, this is not the case in cells containing *w*Au which show no signs of cholesterol trapping, and the addition of 2HPCD is not able to rescue ZIKV infection in these cells [12,13]. In this study, the addition of 2HPCD to cells containing *w*Mel did not result in the rescue of either SFV infection or ONNV infection, suggesting that releasing trapped cholesterol in these cells is not sufficient to rescue alphavirus replication. The requirement for cholesterol during the entry and fusion of alphaviruses has been well studied in mammalian systems. Both the binding and fusion of several alphaviruses are known to be cholesterol-dependent, and reducing the amount of available cholesterol in cellular membranes via high concentrations of methyl-β-cyclodextrin can significantly reduce the entry of SFV [29]. However, the requirement of lipids/cholesterol during alphavirus replication is not fully understood. Studies have indicated that the accumulation of cholesterol in late endosomes and lysosomes in Niemann–Pick disease A fibroblasts leads to a decrease in viral RNA when these cells are infected with the alphavirus Sindbis virus [30]. Further studies have shown that unlike flaviviruses, which actively increase cholesterol biosynthesis, alphaviruses seem to lead to a decrease in cholesterol biosynthesis: suggesting key differences in their requirement for cholesterol [31,32,33]. Alphaviruses also utilise host cell membranes, namely plasma membranes, in order to form replication complexes, unlike flaviviruses which are known to utilise ER membranes [31,34]. The lipid composition of ER membranes compared to plasma membranes is fundamentally different, and therefore, the effect of *Wolbachia* on the availability of cholesterol at each of these membranes is also likely to be different [35]. Taken together, key differences in cholesterol requirements between alphaviruses and flaviviruses may account for the difference in the ability of 2HPCD to rescue *Wolbachia*-mediated antiviral activity against ONNV and SFV when compared to ZIKV and DENV.

## Data Availability

All raw data can be accessed on the Glasgow University repository https://doi.org/10.5525/gla.researchdata.1572.

## Supplementary Materials

The following supporting information can be downloaded at https://www.mdpi.com/article/10.3390/v16010115/s1, Figure S1: Expression of markers encoded in the viral template in the absence of viral replicase. Cells were transfected with two plasmids, one containing the viral template where FFLuc activity is under the control of the Aedes PUb promoter and SFV genomic promoter and Gluc is under the control of the SFV subgenomic promoter (template) and one empty plasmid as a control. Each graph shows 3 independent experiments carried out in duplicate. Y axis represents relative light units/55,000 cells. Statistical significance was determined via an ordinary one-way ANOVA.
