# Supplementary material for: Evidence of Differences in Cellular Regulation of Wolbachia-Mediated Viral Inhibition between Alphaviruses and Flaviviruses
Source: Viruses. 2024 Jan 13;16(1):115. doi: 10.3390/v16010115 (PMC10818798; doi:10.3390/v16010115)
Supplement: Supplementary file 1 [file viruses-16-00115-s001.zip › viruses-2802671-supplementary.pdf]

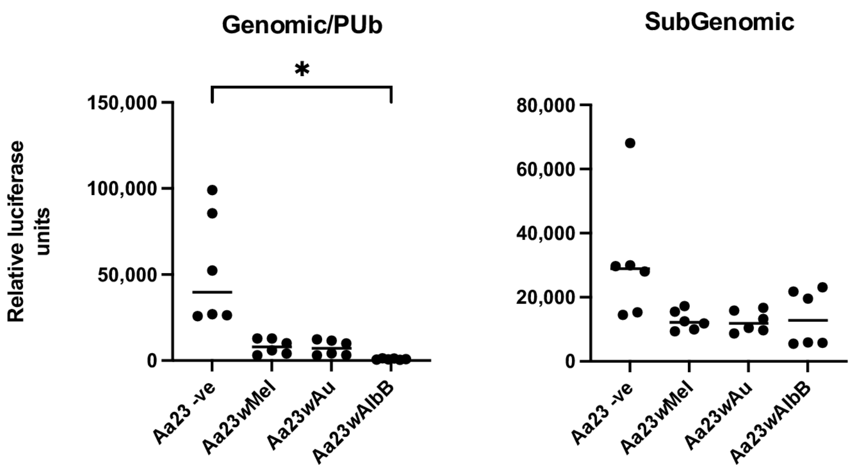

Supplementary Figure S1. Expression of markers encoded in the viral template in the absence of viral replicase. Cells were transfected with two plasmids, one containing the viral template where *FFLuc* activity is under the control of the *Aedes* PUb promoter and SFV genomic promoter and *Gluc* is under the control of the SFV subgenomic promoter (template) and one empty plasmid as a control. Each graph shows 3 independent experiments carried out in duplicate. Y axis represents relative light units/55,000 cells. Statistical significance was determined via an ordinary one-way ANOVA.
